# HPD-Kit: a comprehensive toolkit for pathogen detection and analysis

**DOI:** 10.3389/fcimb.2025.1580165

**Published:** 2025-05-02

**Authors:** Tengcheng Que, Wen Li, Zhining Zhang, Yunlin He, Kangming He, Hong Qiu, Juan Huang, Zhiwei Lu, Chunlan Jiang, Yongjian Huang, Hui Huang, Qiuyu Wu, Panyu Chen, Yanling Hu, Wenjian Liu

**Affiliations:** ^1^ Faculty of Data Science, City University of Macau, Macau, Macau SAR, China; ^2^ School of Basic Medical Sciences, Youjiang Medical University for Nationalities, Baise, Guangxi, China; ^3^ Guangxi Zhuang Autonomous Terrestrial Wildlife Rescue Research and Epidemic Diseases Monitoring Center, Nanning, Guangxi, China; ^4^ Life Sciences Institute, Guangxi Medical University, Nanning, Guangxi, China; ^5^ Department of Biochemistry and Molecular Biology, School of Basic Medicine, Guangxi Medical University, Nanning, Guangxi, China; ^6^ Key Laboratory of Biological Molecular Medicine Research (Guangxi Medical University), Education Department of Guangxi Zhuang Autonomous Region, Nanning, Guangxi, China; ^7^ Guangxi Henbio Biotechnology Co., Ltd., Nanning, Guangxi, China

**Keywords:** pathogen detection, bioinformatics pipeline, HPD-Kit, multi-method alignment, NPAs

## Abstract

**Introduction:**

Unbiased metagenomic sequencing (mNGS) is crucial for infectious disease diagnosis and epidemiological surveillance. However, its analysis requires specialized bioinformatics skills, creating barriers for clinicians. We developed HPD-Kit (Henbio Pathogen Detection Toolkit) with an integrated pathogen database to simplify pathogen detection and analysis for both human and animal pathogens.

**Methods:**

HPD-Kit includes a specifically curated pathogen database and optimized bioinformatics pipeline. We evaluated its performance using simulated datasets at varying pathogen abundances and clinical samples. The toolkit provides both open-source software and a web interface for streamlined one-click analysis.

**Results:**

Validation with simulated data showed HPD-Kit maintains high detection accuracy even at low pathogen abundance. Clinical dataset analysis demonstrated superior pathogen identification compared to conventional methods. The web interface retained this performance while significantly improving usability.

**Discussion:**

HPD-Kit effectively addresses the bioinformatics barrier in mNGS analysis while maintaining high accuracy. Its dual open-source and web-based implementation facilitates clinical and public health applications, promoting wider adoption of mNGS technology in diagnostic settings.

## Highlights

- Three algorithms are used to perform layered alignments, improving detection accuracy.
- The NPAS metric is more effective in identifying dominant pathogens, outperforming unique reads and unique-kmers rankings.
- HPD-Kit supports one-click analysis initiation locally or online, greatly simplifying the process.

## Introduction

The rapid and accurate identification of pathogens after disease onset helps clinicians make early diagnoses and select targeted treatments, ultimately improving patient outcomes and reducing the risk of complications (Land et al., 2018). Pathogen detection is also crucial for public health management, as accurate data can track the spread of infectious diseases, identify outbreak sources, and guide control measures (Chiu and Miller, 2019). However, the diversity of pathogens complicates clinical differentiation, making diagnosis challenging. Studies suggest that up to 60% of infectious cases remain with unidentified causes (Schlaberg et al., 2017). Traditional methods, such as culture and nucleic acid amplification, are time-consuming and often insensitive to certain pathogens. While newer technologies, like amplicon-based tests (e.g., 16S rRNA/18S rRNA), provide faster detection, they are typically limited to specific groups of microorganisms such as bacteria or fungi.

Unbiased metagenomic next-generation sequencing (mNGS) addresses the limitations of these conventional methods by enabling hypothesis-free, culture-independent pathogen detection directly from clinical samples. This approach can identify a broad spectrum of microorganisms, including viruses, bacteria, fungi, and parasites, and it often detects pathogens that traditional methods fail to identify (Wilson et al., 2014; Naccache et al., 2015; Wilson et al., 2015; Graf Erin et al., 2016; Gu et al., 2016; Chiu et al., 2017; Murkey et al., 2017; Wilson et al., 2017b; Wilson et al., 2017a; Wilson et al., 2018). mNGS has been successfully used to diagnose infections in the central nervous system, bloodstream, respiratory system, digestive tract, and eyes (Hoffmann et al., 2015; Pan et al., 2015; Abril et al., 2016; Zhou et al., 2016; Doan et al., 2017; Gosiewski et al., 2017; Kujiraoka et al., 2017; Pendleton et al., 2017; Wilson et al., 2017a).

However, with the rapid advancement of sequencing technologies, the volume of mNGS data has significantly increased. A key challenge lies in performing accurate and reproducible high-throughput data analysis to extract clinically relevant information for diagnosis and monitoring. Due to its broad-spectrum, mNGS typically generates over 99% host-derived reads (Kostic et al., 2012; Wylie et al., 2012). Therefore, mNGS data analysis requires the removal of host sequences, followed by the alignment of non-host sequences to pathogen reference genomes to estimate the abundance of various taxonomic units. Finally, the likelihood of each pathogen’s involvement in disease must be calculated. Each step involves multiple tools with numerous parameter settings. Researchers must carefully select tools, configure parameters, integrate data, and ensure version compatibility. These decisions can significantly impact the final results and the reproducibility, which is critical for bioinformatics pipelines (Baker, 2016; Suetake et al., 2023).

To ensure the effectiveness, reproducibility, and flexibility of an mNGS data analysis pipeline, it should possess the following key characteristics: (i) sufficient storage and computational resources, with the flexibility to adjust run parameters according to available resources; (ii) a high-quality pathogen reference genome database; (iii) accurate and reproducible bioinformatics pipelines; and (iv) a user-friendly interface or software package. Several software tools for pathogen analysis, such as OneCodex (OneCodex), Sunbeam (Clarke et al., 2019), and SURPI (Naccache et al., 2014), have been developed. However, many of these tools require paid subscriptions or significant computational resources to build the foundational databases and perform analyses.

**Figure 1:**
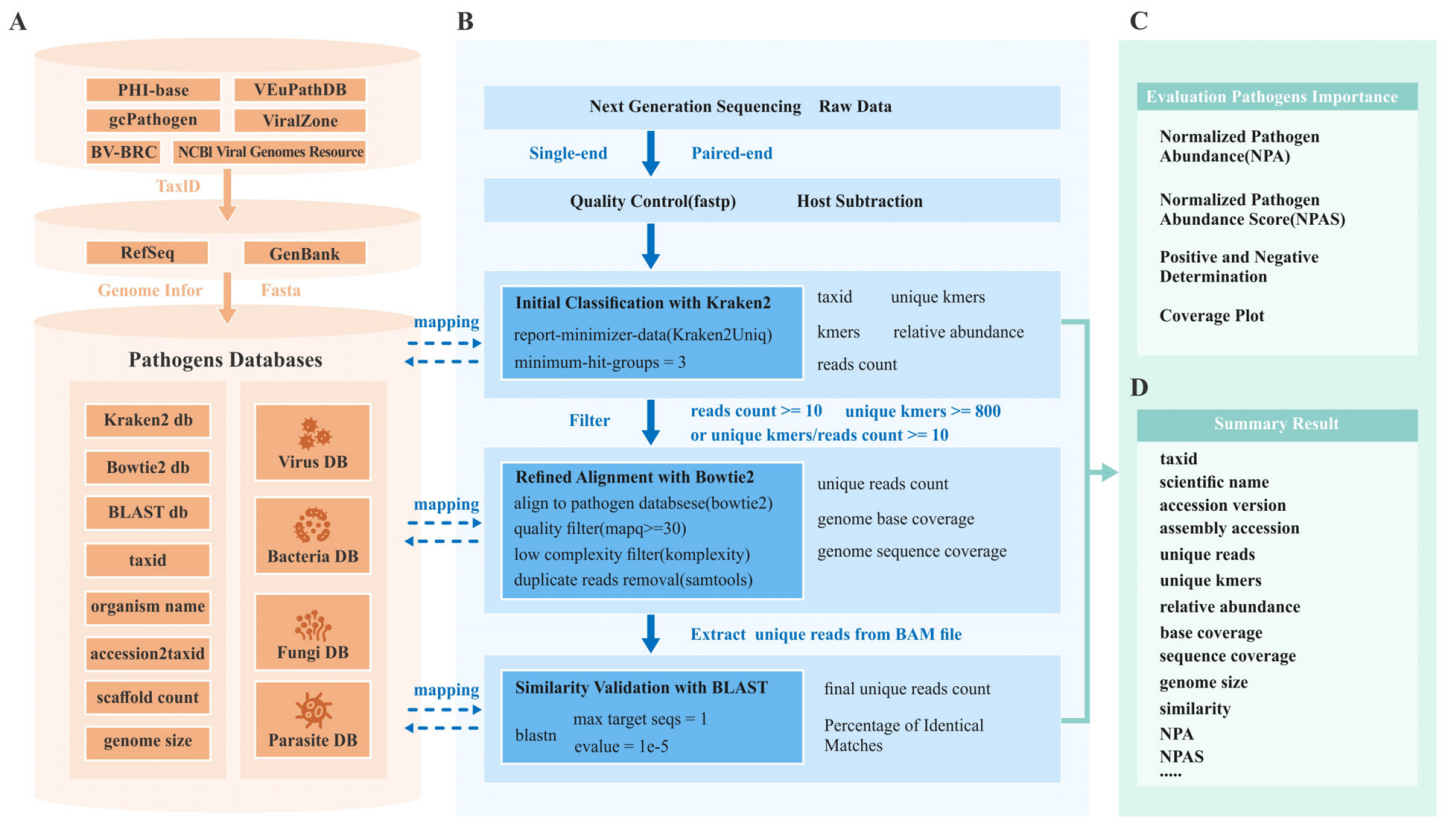
Schematic overview of the workflow of HPD-Kit. **(A)** Overview of the pathogen database construction. **(B-D)** Overview of the pathogen detection workflow of HPD-kit, which includes the following steps: quality control of input data, initial classification using kraken2, refined alignment with bowtie2, similarity validation via BLAST, evaluating of pathogen importance through NPAS scores, and result reports.

To address these challenges, we introduce the Henbio Pathogen Detection Toolkit (HPD-Kit), an open-source, comprehensive tool designed for pathogen detection and analysis in both humans and animals (**Figure 1**). We begin by describing the construction of the pathogen reference genome database. Next, we outline the HPD-Kit bioinformatics pipeline, which includes host subtraction, quality control, multi-method alignment and validation, and pathogen pathogenicity assessment. We then evaluate its pathogen identification capabilities using simulated datasets. Finally, we demonstrate its practical utility through three case studies.

## Method

### Database construction

A comprehensive and non-redundant pathogen reference genome database was constructed through the following steps:

#### Data collection and curation

Pathogen data were first collected from scientific literature and six databases, including the NCBI Virus Database (Brister et al., 2015; Amos et al., 2022; Urban et al., 2022; Olson et al., 2023; Alvarez-Jarreta et al., 2024; Guo et al., 2024). The focus was on pathogens that cause diseases in humans or animals through infection. The collected data encompassed key attributes, including taxonomic ID (TaxID), scientific name, taxonomy, host range, and pathogenicity. Records sharing identical TaxID were consolidated to remove duplicates. For each species (at the species level), reference genome metadata and sequence files were then retrieved and downloaded from NCBI based on the TaxID.

#### Selection of non-redundant reference genomes

To ensure the quality and uniqueness of the reference genomes, priority was given to those from the RefSeq database (Pruitt et al., 2007; O’Leary et al., 2016), which are designated by NCBI as “reference genomes.” For species without records in RefSeq, genome assembly data were sourced from GenBank, prioritizing assembly completeness in the following order: Complete, Chromosome, Scaffold, and Contig (Kitts et al., 2016). To ensure uniqueness, only one genome version per species (defined by TaxID) was retained, with the same criteria applied to different strains.

#### Database construction

Based on the selected non-redundant reference genome data, we constructed a comprehensive pathogen database, categorized into four distinct pathogen types. Each database includes essential information about the pathogens, such as TaxID, genome size, assembly level, and accession number. Additionally, indices and other necessary files were generated using the reference genome sequences, ensuring compatibility with analysis tools like Kraken2 (Lu et al., 2022), Bowtie2 (Langmead and Salzberg, 2012), and BLAST (Johnson et al., 2008). These databases serve as a reliable foundation for the HPD-Kit, facilitating efficient and accurate pathogen identification.

### Bioinformatics pipeline

#### Quality control and host subtraction

Quality control was performed using (version 0.23.4) (Chen et al., 2018) was used for quality control to remove low-quality reads and adapter sequences from the raw data. Reads were discarded if more than 40% of their bases had a quality score below 20, contained over 10 ambiguous bases (N), or were shorter than 30 bases. After quality control, sequences shorter than 80% of their original length were also removed. To reduce host DNA contamination, Bowtie2 (version 2.5.3) (Langmead and Salzberg, 2012) or BBDuk (version 39.08) (BBDuk) was used to align reads to the host reference genome, and only unaligned reads were retained for further analysis.

#### Multiple alignment algorithms and verification

##### Initial classification

The host-subtracted reads were classified using l Kraken2 (version 2.1.3) (Lu et al., 2022) with the parameters –report-minimizer-data and minimum-hit-groups = 3. This step generated TaxIDs, read counts, unique k-mers, and relative abundances for each potential pathogen. To ensure detection sensitivity and minimize false positives, species were retained if they met the following criteria: (i) more than 10 reads, (ii) over 800 unique k-mers, or (iii) a unique k-mer-to-read ratio greater than 10 (i.e., more than 10 unique k-mers per read on average).

##### Refined alignment

Due to homologous sequences and PCR amplification duplicates, the read counts generated by Kraken2 (Lu et al., 2022) may not accurately reflect the true abundance of each pathogen. To obtain non-redundant, high-quality read counts, a refined alignment and quality control were performed. First, Bowtie2 (version 2.5.3) (Langmead and Salzberg, 2012) was used to align the host-subtracted FASTQ files against the reference genome of each pathogen identified by Kraken2, excluding reads with MAPQ < 30 (Lu et al., 2022). Next, Komplexity (version 0.3.6) (Komplexity) was employed to filter sequences with complexity scores < 0.5. Finally, SAMtools (version 1.20) (Danecek et al., 2021) was used to remove duplicate reads and calculated unique read counts, genome base coverage, genome sequence coverage, and sequencing depth for each pathogen.

#### Similarity validation

To assess sequence similarity, the unique reads for each pathogen were aligned using BLAST (version 2.15.0) (Johnson et al., 2008) against the corresponding species reference genome. The top hits (with max_target_seqs = 1 and evalue = 1e^-5^) were selected, and their read counts were used as the final unique read counts for each pathogen. For paired-end reads, only those aligning to the same sequence on the reference genome from both ends were retained.

#### NPAS for identifying infection-related pathogens

Under sufficient sequencing depth, the presence of a pathogen in a sample should theoretically result in (i) an increase in sequencing reads and k-mer counts proportional to the pathogen’s genome length, (ii) detection of all genome loci, and (iii) adequate coverage of all sequences if the genome contains multiple sequences. Based on these assumptions, we adapted the RPKM (Mortazavi et al., 2008) normalization method, commonly used in transcriptomics, to define a new metric: Normalized Pathogen Abundance (NPA). This metric is designed to quantify and evaluate pathogen abundance in a sample by integrating the counts of unique kmers, unique reads, and the size of the pathogen reference genome. The NPA for each pathogen in the sample is calculated as follows (Equation 1):


(1) $$\begin{array}{l} NPA\;=\;\frac{unique\_kmers\;\times \;unique\_reads}{\frac{genome\_size}{1000}\;\times \;\frac{sample\_total\_reads}{1000000}}\\ \begin{array}{c} =\;\frac{unique\_kmers\;\times \;unique\_reads}{genome\_size\times \;sample\_total\_reads}\;\times \;10^{9} \end{array} \end{array}$$

Where *unique_kmers* and *unique_reads* represent the number of unique k-mers and unique reads detected for a given species in the sample, respectively; *genome_size* refers to the size of the species’ reference genome; and *sample_total_reads* indicates the total number of reads in the sample after host subtraction.

Subsequently, the importance or pathogenic potential of each pathogen in the sample can be assessed using the Normalized Pathogen Abundance Score (NPAS), calculated as follows (Equation 2):


(2) $$\begin{array}{c} NPAS\;=\;{\text{log}}_{2}\left(\frac{NPA_{\text{treat}}}{NPA_{\text{control}}\;+\;1}\;\times \;base\_coverage\;\times \;sequence\_coverage\;+1\right) \end{array}$$

**Figure 2:**
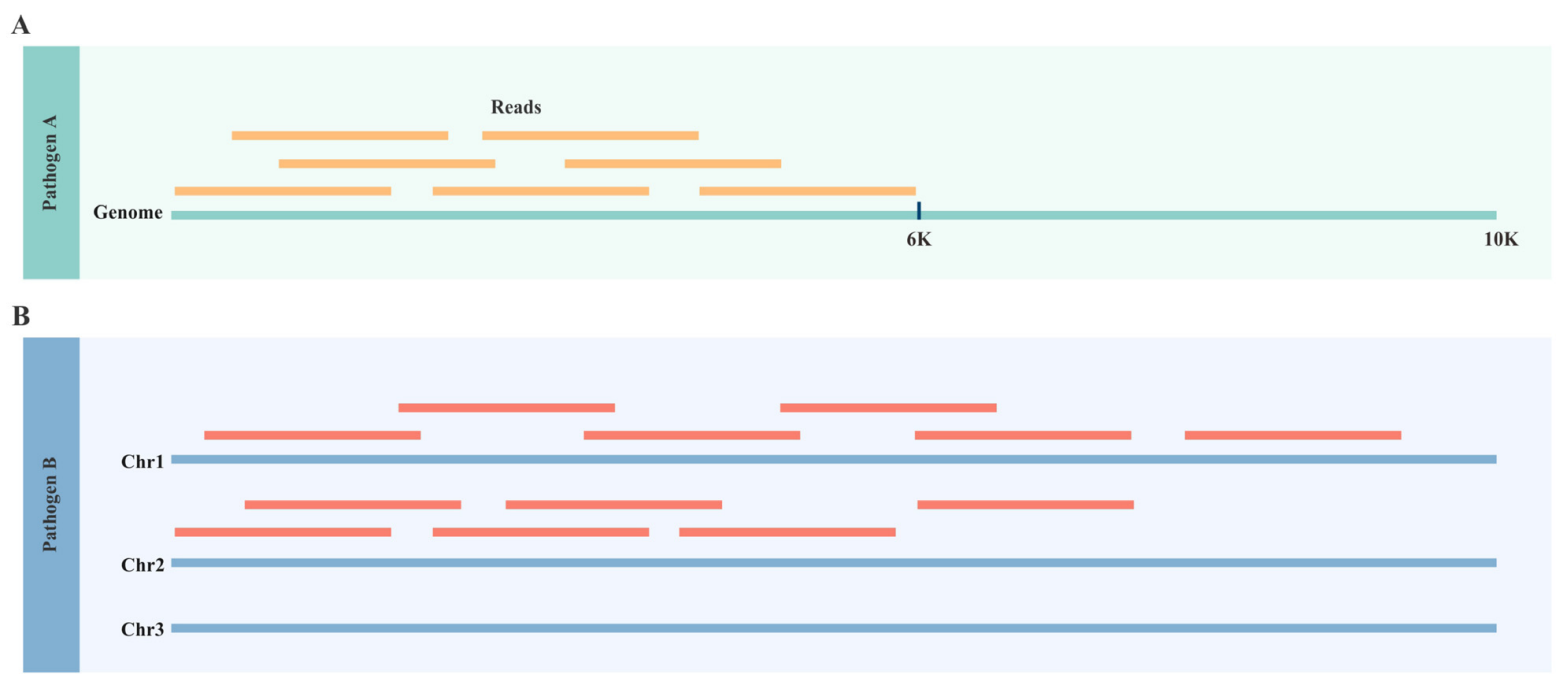
Definition of “Coverage” Metrics. **(A)** The *base_coverage* metric represents the percentage of base pairs in the reference genome covered by sequencing reads, regardless of coverage depth. For example, Pathogen A’s reference genome is 10,000 bp long, and 6,000 bp are covered by sequencing reads, the *base coverage* is 60.0%. **(B)** The *sequences_coverage* metric represents the percentage of sequences in the reference genome covered by sequencing reads, regardless of base coverage. For example, Pathogen B’s reference genome contains 3 sequences, and 2 are covered by sequencing reads, the *sequence_coverage* is 66.7%.

Where *NPA*_treat_ and *NPA*_control_ represent the NPA values in the case and control samples, respectively; *base*_*coverage* refers to the base-level coverage, reflecting the extent of coverage of reference genome positions by the reads in the sample (**Figure 2A**); *sequences_coverage* refers to the sequence-level coverage, indicating the proportion of the reference genome sequences covered by the reads in the sample (**Figure 2B**). A log2 transformation is applied to normalize the data distribution, and adding 1 prevents extreme values from ratio calculation and log2 transformation.

When a pathogen is present in both case and control samples, dividing by (*NPA*_control_ + 1) reduces the pathogen’s significance in the case sample. This approach prioritizes pathogens that are truly associated with infection based on their NPAS scores. If *NPA*_control_ equals 0 (i.e., the pathogen is absent in the control sample) or is close to 0, the NPAS formula simplifies to (Equation 3):


(3) $$\begin{array}{c} NPAS\;=\;{\text{log}}_{2}\left(NPA_{\text{treat}}\;\times \;base\_coverage\;\times \;sequence\_coverage\;+1\right) \end{array}$$

All detected potential pathogens in the sample are then ranked by their NPAS scores, with higher scores indicating a greater likelihood that the pathogen is dominant and related to the disease infection. It is recommended to submit the top ten pathogens to pathologists and clinicians for further validation using independent methods, in conjunction with the clinical symptoms of the case.

### Local analysis workflow construction

Pathogen identification is a complex analytical process that involves multiple steps and software tools, often complicated by compatibility issues between different software versions. To simplify this process and reduce the technical barriers, we adopted containerization technology alongside the Nextflow workflow framework (Di Tommaso et al., 2017). This approach enabled us to package the pathogen identification pipeline into a robust, plug-and-play, and reproducible tool. First, we utilized Singularity (now known as Apptainer) (Apptainer) to bundle all the required software tools into a single container image. Next, we employed Nextflow to script the entire pathogen identification and analysis workflow, transforming it into a user-friendly command-line tool. Customizable parameters enhance the tool’s versatility and flexibility, catering to diverse user needs.

The pipeline supports both single-end and paired-end high-throughput sequencing files and allows for the parallel processing of multiple samples. The system dynamically adjusts the number of parallel samples based on available server resources, significantly improving processing efficiency. Users only need to download the prepackaged Singularity image and the associated pathogen database, set up the Singularity environment, and provide FASTQ files along with a few essential parameters. With a single command, they can execute the entire pathogen analysis pipeline, quickly obtaining comprehensive results on potential pathogens present in the samples.

### Online analysis workflow construction

Although the local analysis workflow greatly simplifies the pathogen identification process, it may still present challenges for users lacking experience with Linux operating systems or sufficient computational resources. To address this, we developed an online pathogen identification and analysis module based on the bioinformatics cloud platform HiOmics (Li et al., 2024). We first adopted Docker (Docker: Accelerated, Containerized Application Development) container technology to package all necessary tools and dependencies into a lightweight container image. Next, we scripted the detailed workflow using the Workflow Description Language (WDL) (OpenWDL: Community Driven Open-development Workflow Language), ensuring clarity and maintainability. Finally, we employed Cromwell (Cromwell: A Workflow Management System) as the execution and scheduling engine to fully automate the process.

Users are not required to download or install any software or databases. Instead, they can simply upload FASTQ files through the Web interface, configure a few essential parameters, and initiate the pathogen analysis workflow with a single click. This cloud-based solution removes the technical burden, making it accessible to a broader range of users, including those without advanced computational expertise.

## Results

### High-quality, non-redundant pathogen reference genome database

**Figure 3:**
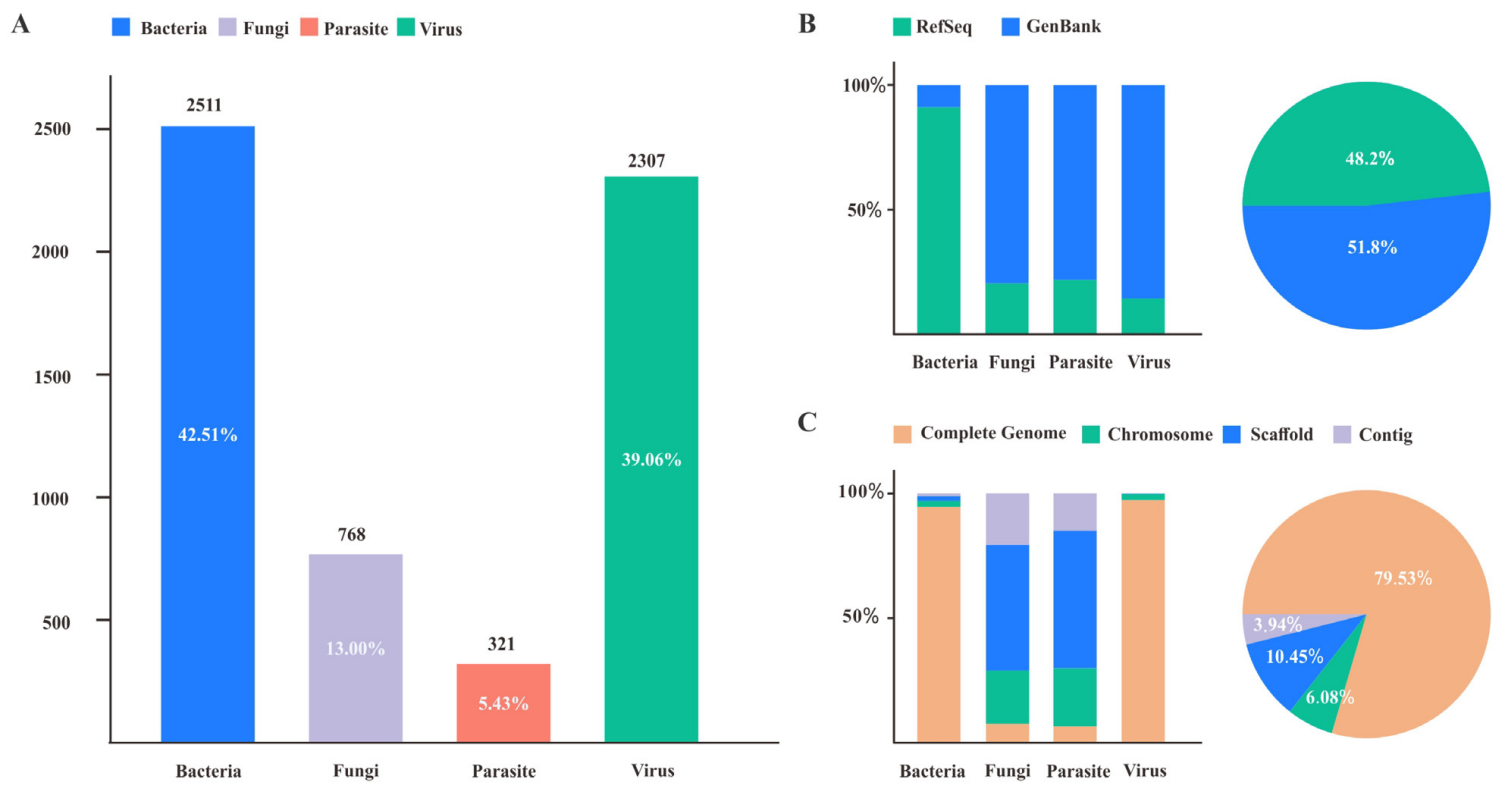
Overview of pathogen reference genomes. **(A)** The number and proportion of bacteria, fungi, parasites, and viruses in the database; **(B)** The sources of reference genomes; **(C)** The completeness of reference genomes.

After rigorous screening, we compiled a comprehensive database of nearly 6,000 entries of reference genome data for pathogens, (**Figure 3**) which include 2,409 bacterial species, 768 fungal species, 2,307 viral species, and 321 parasitic species (as of September 20, 2024). Each species is represented by a single, high-quality, non-redundant reference genome, ensuring consistency and reliability for downstream analyses and pathogen identification.

### Evaluation on simulated datasets

To evaluate the accuracy of HPD-Kit in pathogen identification, we randomly selected two species each from viruses, bacteria, fungi, and parasites, in addition to the reference genome of the human host. Paired-end test datasets were generated using the wgsim (Li et al., 2009) sequencing simulator (version 1.20) from SAMtools, with a read length of 100 bp and an error rate of 1%.

**Figure 4:**
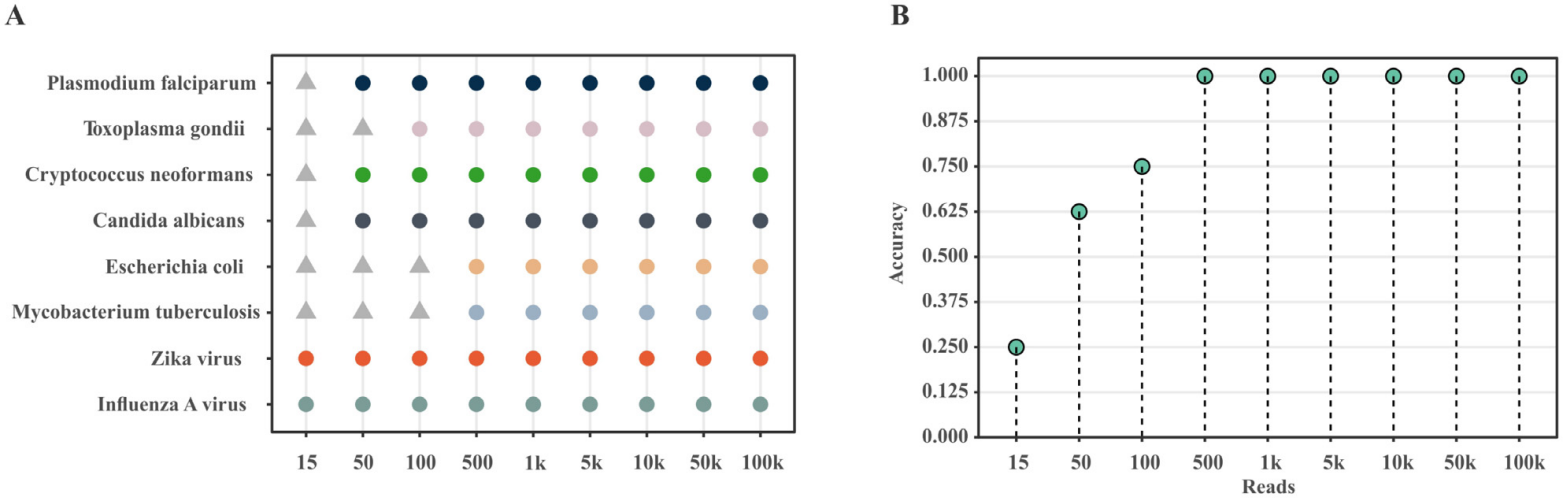
Evaluation results on simulated datasets. **(A)** Detection outcomes of various species at different abundances. The x-axis represents read count, and the y-axis represents randomly selected parasitic, fungal, bacterial, and viral species. Circles indicate detected species, while triangles indicate undetected species. **(B)** Overall detection accuracy of pathogens at different abundances.

To determine the impact of pathogen abundance (read count) in the sample on the detection accuracy of HPD-Kit, we generated nine benchmark datasets with varying read counts: 15, 50, 100, 500, 1,000, 5,000, 10,000, 50,000, and 100,000. In each dataset, the human host read count was fixed at 1 million. As illustrated in **Figure 4**, when the read count of ≥500, the detection accuracy of HPD-Kit for viruses, bacteria, fungi, and parasites achieved 100%. Even at a low read count of 50, the overall accuracy remained at 62.5%. d These results demonstrate that HPD-Kit maintains robust detection performance even under conditions of low pathogen abundance. Notably, the detection accuracy for viruses remained consistently at 100%, across all nine abundance levels.

For pathogens with a large number of reads and k-mers after initial classification, if detailed alignment and similarity validation yield only a small number of unique reads, HPD-Kit flags potential false positives (see **Table 1**). Further analysis confirmed that these pathogens were indeed absent from the simulated dataset.

**Table 1:** Results of Multiple Alignment Algorithms with a Read Count of 500 in Simulated Dataset.

TaxID	ScientificName	PathogenType	Kraken2				Bowtie2			BLAST		False Positive
			Reads	Kmers	Unique Kmers	Relative Abundance	Unique Reads	Base Coverage	Sequence Coverage	Unique Reads	Similarity	
864142	Plasmodium ovale wallikeri	parasite	3741	36442	5521	0.776	4	0.0018	0.16	2	99	Yes
5855	Plasmodium vivax	parasite	15591	50599	5481	3.234	0	0	0	0	0	Yes
1810908	Aspergillus spinulosporus	fungi	33684	170659	15759	6.988	2	7.00E-04	0.02	1	97.5	Yes
2822231	Phanerodontia chrysosporium	fungi	5240	29433	2432	1.087	0	0	0	0	0	Yes
41061	Aspergillus nomiae	fungi	649	5473	906	0.135	0	0	0	0	0	Yes
308745	Aspergillus rambellii	fungi	2277	12418	822	0.472	0	0	0	0	0	Yes

### Applications in clinical datasets

To evaluate the performance of HPD-Kit on clinical data, we replicated key findings from three published studies focusing on infectious diseases affecting the human digestive, visual and nervous systems. Consistent results were obtained using both local software packages and cloud-based workflows, demonstrating HPD-Kit’s reproducibility and effectiveness in pathogen identification across diverse disease contexts. Additionally, researchers can use HiOmics’ visualization plugin to generate heatmaps, coverage maps, scatter plots, and other publication-ready visualizations by inputting the results files from the pathogen identification workflow.

#### Application 1: pathogen identification in diarrhea

**Figure 5:**
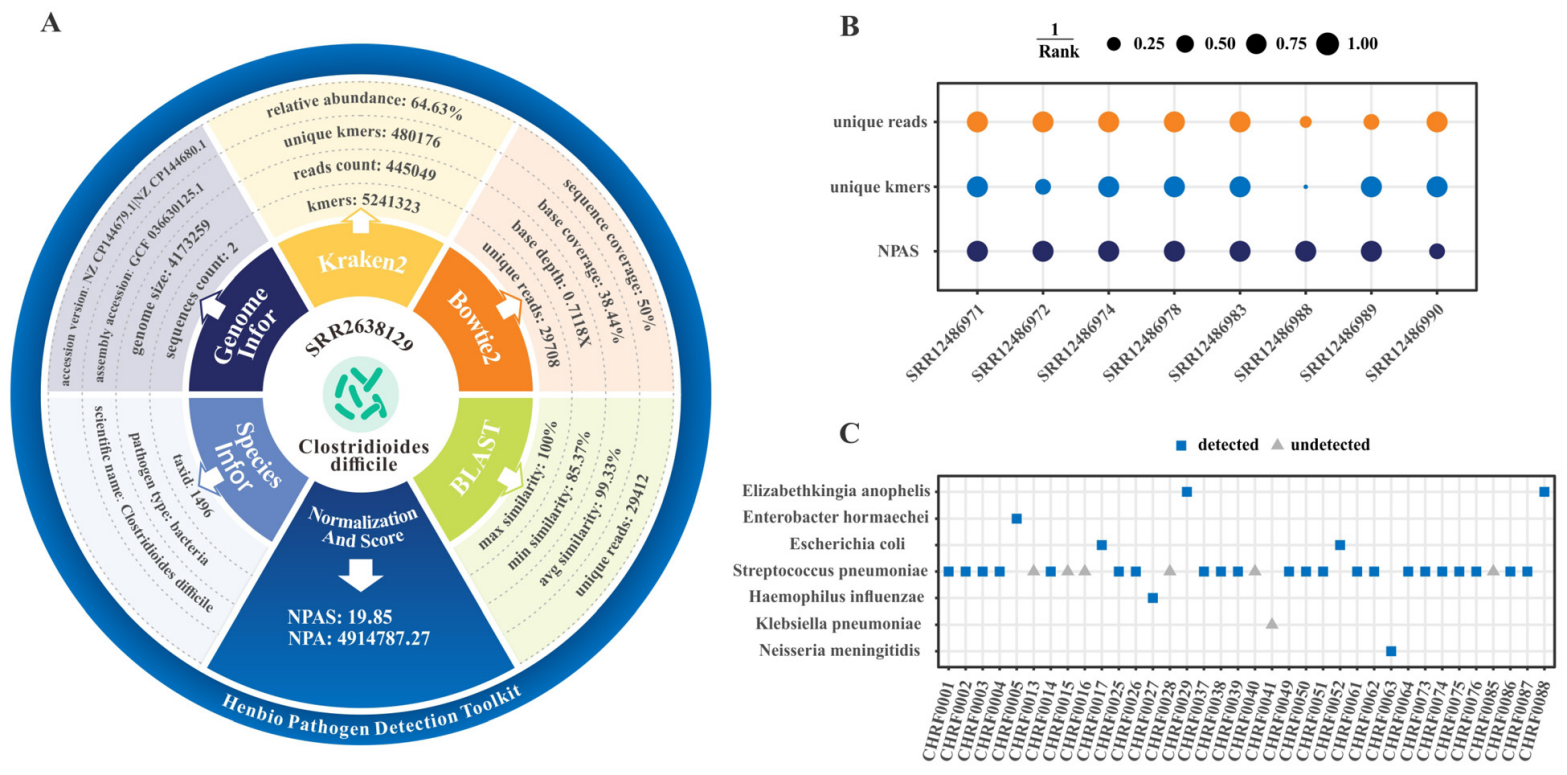
Pathogen identification results by HPD-Kit in real datasets. **(A)** Detection summary of *Clostridium difficile* in sample SRR2638129 from the diarrhea dataset. **(B)** Unique reads, unique k-mers, and NPAS rankings of pathogens in 8 cases of infectious keratitis. Dot size represents the inverse rank; the largest dot indicates the highest rank (rank 1). NPAS rankings more effectively prioritize infection-related pathogens compared to unique reads or unique k-mers. **(C)** Pathogen detection results in 36 meningitis samples. Blue squares denote pathogens detected by HPD-Kit, while gray triangles indicate undetected pathogens.

The HPD-Kit successfully replicated the findings of Yanjiao Zhou et al. (2016). by detecting *Clostridium difficile* in all qPCR-positive samples (**Supplementary Table S1**), demonstrating its advantage in pathogen detection without prior knowledge. **Figure 5A** shows the analysis results for *Clostridium difficile* in sample SRR2638129, highlighting that HPD-Kit not only identifies pathogens but also provides detailed information for a comprehensive understanding of their characteristics.

#### Application 2: pathogen identification in infectious keratitis

In a previous study (Li et al., 2018), Jennifer Lu et al. introduced a workflow using Kraken (Lu et al., 2022) for pathogen identification and Pavian (Breitwieser and Salzberg, 2020) for interactive analysis of metagenomic data from infectious keratitis patient samples. We reanalyzed this dataset using HPD-Kit and obtained similar results. **Figure 5B** summarizes the pathogen identification outcomes for 10 samples (8 infectious keratitis cases and 2 controls), presenting read counts, k-mers distributions, and NPAS scores as heatmaps. Notably, in sample SRR12486990 (**Supplementary Table S2**), *Staphylococcus aureus* ranked second in NPAS score, while *Staphylococcus argenteus* ranked first. Although *S. aureus* exhibited higher abundance in the case sample, its presence in both control samples resulted in a slightly lower NPAS score compared to *S. argenteus*. In contrast, the original study ranked *S. aureus* first based on its z-score. Both *S. aureus* and *S. argenteus* belong to the *Staphylococcus* genus, are Gram-positive, and share similar morphology. Prior to 2015, *S. argenteus* was classified as a subspecies of *S. aureus* (Zhang et al., 2016), suggesting that further differentiation between these two organisms may require additional experimental validation.

#### Application 3: pathogen identification in meningitis

In a study (Saha et al., 2019) involving 36 meningitis cases with known etiologies, Senjuti et al. successfully identified pathogens in 25 cases (69.4%) using a pathogen-calling algorithm based on IDseq. In comparison, HPD-Kit identified pathogens in 29 cases (80.6%) without requiring control samples (**Figure 5C**; **Supplementary Table S3**). Among these, the NPAS score ranked the causative pathogen first in 22 cases, second in one case (CHRF0050, undetected by the original method), third in three cases, and fourth in two cases (CHRF0039, also undetected). One case ranked seventh (CHRF0001) and another eighth (CHRF0004), both of which were missed by the original method. Additionally, HPD-Kit also identified three cases of *neuroinvasive chikungunya virus* (CHIKV), confirming a previously unrecognized meningitis outbreak.

## Discussion

Pathogen detection is increasingly recognized as a critical tool for improving healthcare quality and safeguarding public health (Armstrong Gregory et al., 2019). However, converting raw FASTQ files into pathogen identification results remains a complex task, especially for clinicians lacking bioinformatics expertise. To address this challenge, we developed HPD-Kit, a bioinformatics pipeline specifically designed for human and animal pathogen detection. HPD-Kit streamlines the processing of high-throughput sequencing data, enabling users to perform comprehensive analyses without requiring programming skills. It offers both a local software package and an online analysis platform that deliver accurate and reproducible results.

The use of reference genomes provides a reliable framework of genetic information for pathogen detection, significantly enhancing both accuracy and efficiency (Kaye and Wasserman, 2021). In our study, we constructed a curated database comprising key pathogens that infect humans and animals, while excluding the majority of non-pathogenic microorganisms to minimize potential noise. For each species, we selected the most complete and highest-quality reference genome currently available. Unlike other tools that rely on broad-spectrum microbial genome databases, such as the NCBI RefSeq employed by SURPI (Naccache et al., 2014), GATK (Walker et al., 2018), and Kraken (Lu et al., 2022), or nucleotide (nt) and non-redundant protein (nr) databases like IDseq (Kalantar et al., 2020), HPD-Kit’s pathogen-specific database minimizes computational overhead and reduces interference from non-pathogenic microbes.

A common challenge faced by many microbial identification tools is the frequent reporting of false positives due to low-abundance reads. To reduce false positive rates, Lu et al. recommend applying stringent filters (reads > 10 & unique k-mers > 1000) when using Kraken2 for pathogen identification (Lu et al., 2022). However, this approach risks filtering out some truly present low-abundance pathogens. For instance, in samples SRR3214089 (total reads: 769; reads of Epstein-Barr virus: 15; unique k-mers of Epstein-Barr virus: 557) and SRR3214092 (total reads: 25050; reads of JC polyomavirus: 8067; unique k-mers of JC polyomavirus: 883), both Epstein-Barr virus and JC polyomavirus would be incorrectly classified as false positives. To strike a balance between reducing false positives and enhancing the detection of low-abundance pathogens, HPD-Kit integrates three complementary algorithms: Kraken2 (Lu et al., 2022), Bowtie2 (Langmead and Salzberg, 2012), and BLAST (Johnson et al., 2008). Since Kraken2 is used only for initial screening in HPD-Kit, we adjusted its filter criteria to unique k-mers > 800 or unique k-mers-to-read ratio > 10. This adjustment enables the correct detection of pathogens in samples SRR3214089 and SRR3214092. Testing on simulated datasets demonstrated that HPD-Kit achieved an identification accuracy rate of 62.5% even with a low number of reads (50), a critical capability for diagnosing and treating diseases with early-stage low pathogen abundance. **Table 1** further highlights HPD-Kit’s effectiveness in reducing false positives.

Accurately identifying the true infectious agent from a large pool of potential candidates remains a significant challenge in pathogen analysis. To address this, HPD-Kit leverages a rigorously curated pathogen database and integrates multiple key metrics—such as unique reads, unique k-mers, relative abundance, genome coverage, and sequence similarity—to propose The NPAS score, a quantitative measure of the pathogenic potential of candidates. The NPAS score is applicable to both control and non-control sample scenarios. For example, in meningitis cases, HPD-Kit detected more pathogens (independently confirmed by other methods) than original methods (Saha et al., 2019), even when their NPAS scores did not always rank first. Notably, control samples were not used in these specific cases. For clinical diagnostics, we strongly recommend the inclusion of control samples to minimize the potential influence of pathogens present in controls on NPAS scoring.

In the context of public health surveillance, HPD-Kit holds significant potential, particularly in its capacity for rapid response during infectious disease outbreaks. Its streamlined bioinformatics workflow and user-friendly interface enable the efficient processing of high-throughput sequencing data, providing public health laboratories with a powerful tool. Furthermore, its curated database and filtering algorithms are likely to maintain high accuracy in complex samples, thereby supporting the differentiation of outbreak strains from background microbial communities. By reducing the time and expertise required for pathogen detection, HPD-Kit has the potential to enhance the ability of public health systems to effectively respond to emerging infectious disease threats.

Although our pathogen database covers the majority of known pathogens, the HPD-Kit is currently unable to detect newly emerging pathogens that are not yet included. To address this limitation, we have implemented the following measures: Users can report missing or newly discovered pathogens via the email provided on GitHub, and our team will review and update the database within one month. Additionally, we conduct a comprehensive review of updates from databases such as NCBI Virus Database every six months and promptly upgrade the HPD database to ensure the inclusion of newly emerging pathogens. In response to emergencies (e.g., disease outbreaks), we act swiftly to update and release new versions of the database, ensuring that users always have access to the most up-to-date data. Through these measures, we have significantly enhanced the timeliness and comprehensiveness of the database.

In the future, we plan to further enhance the functionality of HPD-Kit by integrating pathogen genome assembly and mutation analysis into its bioinformatics pipeline, leveraging artificial intelligence to identify and characterize pathogen marker genes, and improving the quality of microbial draft genomes to reduce false-positive rates in microbial identification. These advancements aim to transform HPD-Kit into a more powerful and versatile tool for pathogen identification and analysis.

## Conclusion

In summary, HPD-Kit offers an efficient and user-friendly solution for pathogen analysis and identification, facilitating the broader adoption of bioinformatics tools in pathogen detection. Users can choose between a local software package or a web-based interface, requiring minimal parameter adjustments to complete analyses. This approach not only empowers researchers with limited programming experience but also provides clinicians with a reliable and accurate method for pathogen identification, thereby supporting disease diagnosis and public health decision-making.

## Data Availability

The datasets presented in this study can be found in online repositories. The names of the repository/repositories and accession number(s) can be found in the article/**Supplementary Material**.
