# Supplementary material for: HPD-Kit: a comprehensive toolkit for pathogen detection and analysis
Source: Front Cell Infect Microbiol. 2025 May 2;15:1580165. doi: 10.3389/fcimb.2025.1580165 (PMC12081367; doi:10.3389/fcimb.2025.1580165)
Supplement: Supplementary file 1 [file Table1.docx]

Table S1. Pathogen detection results using HPD-Kit in diarrhea cases

| **SampleID** | **Clostridioides difficile** | **Norovirus** | **Sapovirus** |
| --- | --- | --- | --- |
| SRR2638109 | 1 | 0 | 0 |
| SRR2638110 | 1 | 0 | 0 |
| SRR2638111 | 1 | 0 | 1 |
| SRR2638112 | 1 | 0 | 0 |
| SRR2638113 | 1 | 0 | 0 |
| SRR2638114 | 1 | 1 | 0 |
| SRR2638115 | 1 | 1 | 0 |
| SRR2638116 | 1 | 0 | 0 |
| SRR2638117 | 1 | 0 | 0 |
| SRR2638118 | 1 | 0 | 0 |
| SRR2638119 | 1 | 0 | 0 |
| SRR2638120 | 1 | 0 | 0 |
| SRR2638121 | 1 | 0 | 0 |
| SRR2638122 | 1 | 1 | 0 |
| SRR2638124 | 1 | 1 | 0 |
| SRR2638125 | 1 | 0 | 0 |
| SRR2638126 | 1 | 0 | 0 |
| SRR2638127 | 1 | 1 | 0 |
| SRR2638129 | 1 | 0 | 1 |
| SRR2638131 | 1 | 0 | 0 |
| SRR2638133 | 1 | 0 | 0 |
| SRR2638136 | 1 | 0 | 0 |
| Note: 1 = detected, 0 = not detected. The red number 1 indicates a false positive. | | | |

Table S2. Pathogen detection results using HPD-Kit in infectious keratitis case

| **Sample** | **Expected Pathogen** | **PathogenType** | **NPAS** | **NPAS Rank** | **Max NPAS Pathogen** |
| --- | --- | --- | --- | --- | --- |
| SRR12486971 | Anncaliia algerae | fungi | 17.360 | 1 | Anncaliia algerae |
| SRR12486972 | Aspergillus flavus | fungi | 12.447 | 1 | Aspergillus flavus |
| SRR12486974 | Candida albicans | fungi | 13.310 | 1 | Candida albicans |
| SRR12486978 | Mycobacteroides chelonae | bacteria | 24.140 | 1 | Mycobacteroides chelonae |
| SRR12486983 | Human alphaherpesvirus 1 | virus | 20.970 | 1 | Human alphaherpesvirus 1 |
| SRR12486988 | Acanthamoeba castellanii | parasite | 0.388 | 1 | Acanthamoeba castellanii |
| SRR12486989 | Streptococcus agalactiae | bacteria | 16.761 | 1 | Streptococcus agalactiae |
| SRR12486990 | Staphylococcus aureus | bacteria | 11.273 | 2 | Staphylococcus argenteus |

Table S3. Pathogen detection results using HPD-Kit in meningitis case

| **Sample** | **Expected Pathogen** | **HDP-Kit Result** | **NPAS** | **NPAS Rank** |
| --- | --- | --- | --- | --- |
| CHRF0001 | Streptococcus pneumoniae | 1 | 1.322 | 7 |
| CHRF0002 | Streptococcus pneumoniae | 1 | 25.741 | 1 |
| CHRF0003 | Streptococcus pneumoniae | 1 | 6.836 | 3 |
| CHRF0004 | Streptococcus pneumoniae | 1 | 0.436 | 8 |
| CHRF0005 | Enterobacter hormaechei | 1 | 15.874 | 1 |
| CHRF0013 | Streptococcus pneumoniae | 0 | - | - |
| CHRF0014 | Streptococcus pneumoniae | 1 | 7.375 | 1 |
| CHRF0015 | Streptococcus pneumoniae | 0 | - | - |
| CHRF0016 | Streptococcus pneumoniae | 0 | - | - |
| CHRF0017 | Escherichia coli | 1 | 8.632 | 1 |
| CHRF0025 | Streptococcus pneumoniae | 1 | 9.873 | 1 |
| CHRF0026 | Streptococcus pneumoniae | 1 | 22.232 | 1 |
| CHRF0027 | Haemophilus influenzae | 1 | 13.376 | 1 |
| CHRF0028 | Streptococcus pneumoniae | 0 | - | - |
| CHRF0029 | Elizabethkingia anophelis | 1 | 15.861 | 1 |
| CHRF0037 | Streptococcus pneumoniae | 1 | 12.525 | 1 |
| CHRF0038 | Streptococcus pneumoniae | 1 | 7.172 | 1 |
| CHRF0039 | Streptococcus pneumoniae | 1 | 0.127 | 4 |
| CHRF0040 | Streptococcus pneumoniae | 0 | - | - |
| CHRF0041 | Klebsiella pneumoniae | 0 | - | - |
| CHRF0049 | Streptococcus pneumoniae | 1 | 2.198 | 4 |
| CHRF0050 | Streptococcus pneumoniae | 1 | 5.827 | 2 |
| CHRF0051 | Streptococcus pneumoniae | 1 | 20.066 | 1 |
| CHRF0052 | Escherichia coli | 1 | 20.371 | 1 |
| CHRF0061 | Streptococcus pneumoniae | 1 | 13.339 | 1 |
| CHRF0062 | Streptococcus pneumoniae | 1 | 17.598 | 1 |
| CHRF0063 | Neisseria meningitidis | 1 | 25.260 | 1 |
| CHRF0064 | Streptococcus pneumoniae | 1 | 24.927 | 1 |
| CHRF0073 | Streptococcus pneumoniae | 1 | 24.622 | 1 |
| CHRF0074 | Streptococcus pneumoniae | 1 | 7.438 | 3 |
| CHRF0075 | Streptococcus pneumoniae | 1 | 13.466 | 1 |
| CHRF0076 | Streptococcus pneumoniae | 1 | 14.537 | 1 |
| CHRF0085 | Streptococcus pneumoniae | 0 | - | - |
| CHRF0086 | Streptococcus pneumoniae | 1 | 14.319 | 1 |
| CHRF0087 | Streptococcus pneumoniae | 1 | 0.831 | 3 |
| CHRF0088 | Elizabethkingia anophelis | 1 | 12.570 | 1 |
| Note: Gray font indicates pathogens not identified in the original mNGS data analysis.  In the “HDP-Kit Result” column, 1 = detected, 0 = not detected. | | | | |
